# Children Understand How Adults’ Achievement Goals Drive Actions

**DOI:** 10.1162/OPMI.a.331

**Published:** 2026-02-10

**Authors:** Brandon A. Carrillo, Mika Asaba, Julia A. Leonard

**Affiliations:** Department of Psychology, Yale University, New Haven, CT, USA

**Keywords:** achievement goals, social cognition, competence

## Abstract

Adults often hold different goals for children’s achievement: Sometimes they want a child to learn and develop their skills as much as possible (i.e., a learning goal), while other times they may forego a child’s learning in favor of successful performance (i.e., a performance goal). How do children think these achievement goals influence adults’ child-directed behaviors? Across two preregistered experiments (*n* = 90 adults; *n* = 160 5- to 8-year-old children), we found that children systematically predict that an adult would select a more difficult task for a recipient child when the adult held a learning (vs. performance) goal, and when the recipient was more (vs. less) competent. Importantly, we found that this pattern matched adults’ actual task choices, although adults showed more sensitivity to choosing a task that anchors closely to what a child can reasonably learn from or accomplish. These results suggest children can reason about how adult’s achievement goals manifest into observable actions, which may have consequences for children’s own goal orientations and task selections.

## INTRODUCTION

Achievement goals shape children’s learning and motivation (Elliot & Hulleman, 2017; Elliott & Dweck, 1988; Grant & Dweck, 2003). In classic models, these goals have been classified as either a desire to develop new knowledge and skills (learning goal) or a desire to prove one’s competence and/or avoid appearing incompetent (performance goal; Elliott & Dweck, 1988). Decades of correlational and experimental work have shown that children who want to learn, rather than perform, are more likely to persist, seek challenges, maintain positive beliefs about the self in the face of setbacks, and have greater academic achievement (Ames & Archer, 1988; Elliott & Dweck, 1988; Grant & Dweck, 2003; Huang, 2012; Meece et al., 1988; Scherrer et al., 2020). This body of work has been highly influential, forming the foundation of research on growth mindset (Dweck & Yeager, 2019; Yeager et al., 2019), inspiring interventions to reduce stereotype threat by focusing on learning goals (Woodcock et al., 2016), and even influencing teacher training and curriculum design (Schiefele & Schaffner, 2015; Shim et al., 2013).

Yet, despite the eminence of work on achievement goals, a basic question remains unanswered: Do children have an abstract understanding of achievement goals that allows them to predict and evaluate others’ actions? We attempt to address this gap by probing children’s reasoning about adults’ achievement goals for children. Indeed, prior work has demonstrated that adults’ achievement goals shape their behaviors toward children (e.g., learning goals lead to more autonomy-supportive parenting; Gonida & Cortina, 2014; Mageau et al., 2016) and influence children’s subsequent learning outcomes (e.g., children of parents with learning goals show higher classroom engagement; Ablard & Parker, 1997; Gonida et al., 2009). However, it is not clear whether children understand, for instance, that an adult with a learning goal would likely select harder tasks (i.e., above a child’s skill level) to encourage growth, whereas an adult with a performance goal would select easier tasks (i.e., tasks at or below the child’s skill level) to ensure success. Here, we test whether children understand how adults’ achievement goals give rise to specific child-directed actions. Answering this question is critical for informing how children interpret pedagogical interactions and learn what adults, like their parents and teachers, value for them.

To illustrate, imagine the following scenario: A parent wants to pick out a book for her child’s upcoming reading contest. If the parent had a performance goal and wanted her child to read a book perfectly and make no mistakes, she would likely pick a book *at* or *below* her child’s reading level. However, if the parent instead had a learning goal and wanted her child to improve their reading skills, she may opt to choose a harder book just *above* her child’s reading level. Critically, this scenario highlights how the parent is not only aware of what she wants her child to achieve (i.e., high performance or skill growth) but that she is sensitive to what her child currently *can* achieve (i.e., her child’s competence). To put it simply, adults’ task choices for children are presumably not only influenced by their specific achievement goals but are also calibrated to a child’s own competence. Here we examine whether adults indeed act this way, as well as whether children as young as 5 or 6, who are at the cusp of formal schooling, understand this causal process.

Prior work shows that children adjust their *own* behavior based on different achievement goals. For example, seminal work by Elliott and Dweck (1988) found elementary school students are more likely to take on challenges, use more adaptive strategies, and show less negative affect when learning (vs. performance) goals are emphasized during a given task. Further, when 5- to 10-year-old children possess performance goals (playing “to win” vs. “for fun”), they selectively make task-relevant parameters easier if given the opportunity (Chu et al., 2025). Beyond these self-directed actions, children adjust their behaviors towards others based on different achievement goals: By age four, children are more likely to demonstrate costly and informative actions to an agent wanting to learn about a toy, as opposed to an agent who simply wants to observe it (Gweon & Schulz, 2019). Additionally, 4- to 6-year-olds give agents who want to learn (vs. complete a task on their own) harder tasks (Jeong & Frye, 2025) and 5- to 7-year-old children pick harder toys for another child who wants to learn and choose easier toys for themselves when they simply want to play (Bridgers et al., 2020). However, it is unknown whether children predict that *other people* will adjust their actions given a specific achievement goal.

It is also unclear whether children think achievement goals will be calibrated to a person’s competence. One possibility is that children simply think that “learning” can be accomplished by doing harder tasks, and “performing” (succeeding), with easier tasks. A second possibility is that children understand that the extent to which “learning” and “performing” can be accomplished via harder and easier tasks depends on their competence. Prior work shows that children are sensitive to others’ competence and expect adults to be as well: 5- and 6-year-old children are more likely to provide exhaustive information about a toy to agents they know lack the appropriate knowledge (Gweon et al., 2018; see also Gweon & Schulz, 2019 and Baer & Odic, 2022; Magid et al., 2018 for work on division of labor and competence) and expect competent students to receive more positive teacher input (e.g., smiling; Brey & Shutts, 2018) but less help than their less capable peers (Sierksma & Shutts, 2020). Furthermore, 5- to 6-year-olds give harder counting tasks to agents who are better (vs. worse) at counting and want to learn (vs. do a task on their own), suggesting that, in their own actions, young children jointly consider an agent’s competence and learning goals (Jeong & Frye, 2025). Given this evidence showing that even preschool-age children rationally account for others’ competence when acting to achieve different goals, we hypothesize that 5- to 6-year-old children will understand that adults with learning (vs. performance) goals will give harder tasks to children and, importantly, will tailor the task difficulty to the receiving child’s competence.

Across two preregistered experiments, we test whether young children can infer how adults’ achievement goals drive their task selection for children. To this end, we created novel paradigms that crossed an adult’s achievement goal (learning vs. performance) with a child’s competence (high vs. low). We decided to use the foundational “learning vs. performance” dichotomy from classic goal orientation research (Ames, 1992; Dweck & Leggett, 1988) as an initial step into understanding children’s intuitions about adults’ achievement goals and child-directed behaviors. Although this binary classification is an oversimplification of contemporary multidimensional frameworks (see Elliot & Hulleman, 2017; Pintrich, 2003 for a review; Bardach et al., 2020; Grant & Dweck, 2003; Hulleman et al., 2010; Senko, 2016; Senko & Dawson, 2017), we view it as a theoretically informative starting point because it allows us to probe whether children are sensitive to the fundamental distinction between learning and performance that underlie these frameworks.

In Experiment 1A, we first establish adults’ actual task choices given different achievement goals and a child’s competence. In Experiment 1B and 1C, we test whether 5- to 6-year-old children predict an adult’s task choice given different achievement goals for children of varying competence (1B) and for themselves (1C). In Experiment 2A, we explore whether adults have expectations about how much harder or easier a task should be to fulfill a learning or achievement goal. In Experiment 2B, we see whether 5- to 8-year-old children hold similar expectations to adults in Experiment 2A (note we extended the age range to explore potential age effects). Across experiments, we used one-shot, familiar, real-world tasks in which children’s competence was either visually observable (tracing) or could be placed on a concrete scale (reading, with reading levels indexing competence). Experiments run with adults were conducted on Amazon Mechanical Turk and all experiments run with children were conducted synchronously with an experimenter on Zoom. Preregistrations for each experiment can be found on OSF: Experiment 1A (https://tinyurl.com/E1A-goals), 1B (https://tinyurl.com/E1B-goals), 1C (https://tinyurl.com/E1C-goals), 2A (https://tinyurl.com/E2A-goals), and 2B (https://tinyurl.com/E2B-goals). All experiment scripts, data, and code can be found on OSF as well (https://tinyurl.com/zmjnf37r).

## EXPERIMENT 1A

In Experiment 1A, we tested whether adults systematically select tasks for children based on their achievement goals and children’s competence.

### Methods

#### Participants.

Forty adults (*M*_*Age*_(*SD*) = 40.72(8.86) years, Range: 25–68) in the United States were recruited via Amazon Mechanical Turk. Participants reported their gender as 63% male, 30% female, and 2% non-binary, with 5% preferring not to answer. Participants reported their race as 83% White, 7% Multiracial, 5% Asian, and 5% Black or African American. An additional two participants were collected but excluded for failing comprehension checks based on preregistered criteria.

#### Materials.

**Figure 1.**
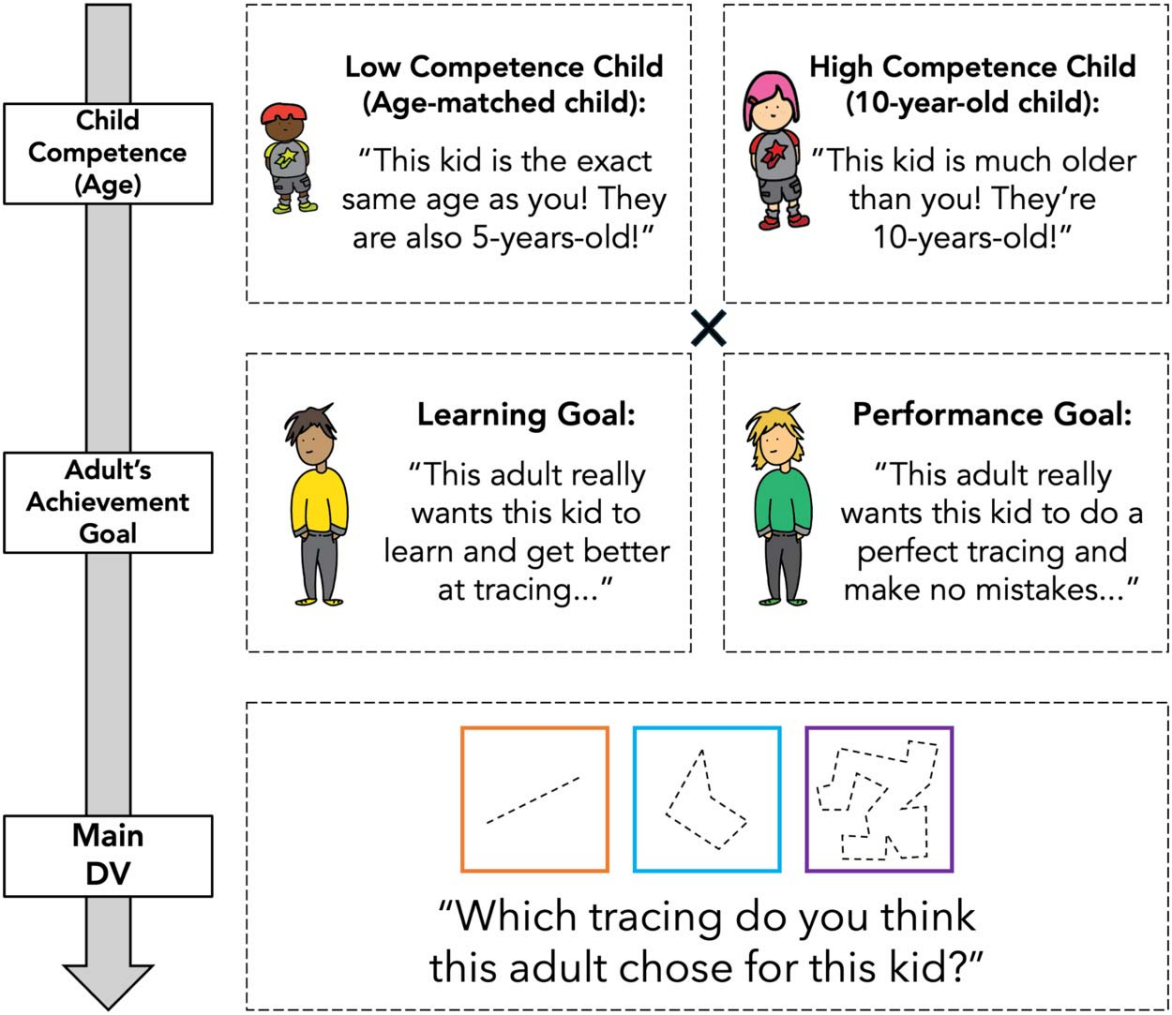
**Schematic of Experiment 1B study design.** Participants saw four total test trials that crossed an adult’s achievement goal (learning vs. performance) with a target child’s competence (low [i.e., age-matched] vs. high [i.e., 10-year-old] competence). Experiment 1A used the same tracings, but did not show the cartoon images of the children and adults.

An online survey was created and administered using the Qualtrics platform. Five unique sets of easy, medium, and hard tracings were created. The easy tracings had 1 line, the medium tracings had 5 lines, and the hard tracings had 21 lines. Easy, medium, and hard tracings were presented in different colored boxes (orange for easy, blue for medium, and purple for hard) and were referred to by these colors (e.g., the orange tracing). See Figure 1 for tracing examples.

#### Procedure.

Participants were first shown three tracings, and asked which tracing was easy, which tracing was medium, and which tracing was hard (*n* = 2 excluded for failing one or more questions).

Next, each participant underwent four test trials. The test trials crossed the provided achievement goal (learning goal vs. performance goal) with the target child’s competence (operationalized via a child’s age: a less competent 5-year-old vs. a more competent 10-year-old). We used age as a marker for competence because we thought it would be easier to understand in this paradigm. Furthermore, we confirmed via pilot testing that 5- and 6-year-old children (our target participant age range in future experiments) expect older children (i.e., 10-year-old children) to be more competent at tracing than younger children (i.e., 5-year-old children; see Supplementary Materials for more details).

For learning goal trials, participants were told that they should want the child to “learn and get better at tracing”. For performance goal trials, participants were told that they should want the child to “do a perfect tracing and make no mistakes”. Participants were asked to select one of the three tracings (easy, medium, or hard; tracings were not explicitly labeled with their difficulty level) for the target child. Trials were blocked by child competence (5-year-old trials first, or 10-year-old trials first) and goal order was counterbalanced within blocks.

### Preregistered Hypotheses and Analysis Plan

We predicted that participants would be more likely to choose harder difficulty tracings for children when they were told to possess a learning goal rather than a performance goal and when the child had either high or low competence.

Across all experiments, we report the maximal model that converged (Barr et al., 2013; full structure of these models described below). Our main model was an ordinal mixed-effects Bayesian regression predicting participants’ tracing choices (1 = easy, 2 = medium, 3 = hard) with fixed effects for achievement goal (learning vs. performance) and child competence (less competent 5-year-old vs. more competent 10-year-old). The model included random slopes for achievement goal and target child competence and random intercepts for participant. In these ordinal models, we report the 95% Credible Intervals (CI) instead of a *p*-value to ascertain the reliability of our main effects. A CI that does not include zero provides sufficient evidence that the effect is significantly different from the null hypothesis.

To interrogate results from our main mixed-effects model, we ran ordinal mixed-effects models looking at the effect of achievement goal for each child competence trial type (less competent 5-year-old child vs. more competent 10-year-old child) and the effect of child competence for each achievement goal trial type (learning vs. performance). This resulted in four ordinal mixed-effects models with fixed effects and random slopes for the predictor variable (i.e., achievement goal or child competence) and random intercepts for participant. We report the results for these regressions for Experiments 1A–C in the Supplementary Materials.

### Results

**Figure 2.**
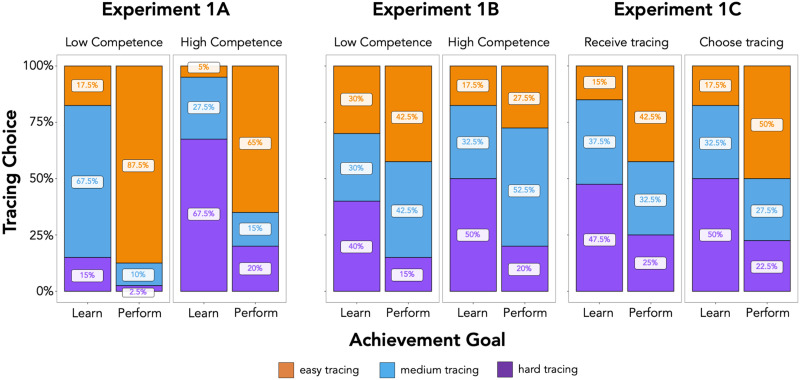
**Results from Experiment 1A (adults) and 1B–C (5- and 6-year-olds).** The *X*-axis shows each achievement goal trial (learning vs. performance) and the *Y*-axis shows the proportion of participants’ tracing choices or predictions. Trials are faceted by the target child’s competence in Experiment 1A (Low vs. High) and Experiment 1B (Low vs. High), and by participant role in Experiment 1C (receive vs. choose). In general, both children and adults chose harder difficulty tracings when the recipient wanted to learn (vs. perform) and was more (vs. less) competent.

As predicted, participants were more likely to choose a harder level of tracing when holding a learning goal versus a performance goal (*b* = −3.76, 95% CI [−6.02, −2.29]) and when the child had high vs. low competence (*b* = 2.00, 95% CI [1.10, 3.33]; see Figure 2). Exploratory analyses revealed that the distribution of participants’ choices differed from chance (33%) across all trials (*X*^2^’s (2, 40) ≥ 18.2, *p*’s < 0.001; Chi-Square Goodness of Fit Test). Specifically, for a less competent 5-year-old, participants were most likely to choose a medium tracing when they had a learning goal (68%, *p* < 0.001; binomial test against chance of 33% with Bonferroni *p*-value correction) and an easy tracing when they had a performance goal (88%, *p* < 0.001). For a more competent 10-year-old, however, participants were most likely to choose a hard tracing when they had a learning goal (68%, *p* < 0.001) and an easy tracing when they had a performance goal (65%, *p* < 0.001). This experiment confirmed that both achievement goals and perceptions of children’s competencies inform adults’ task selections for children.

## EXPERIMENT 1B

Experiment 1A provided the “ground truth” of adults’ task selections based on their achievement goal and the age of the target child (a proxy for competence). In Experiment 1B, we test whether 5- to 6-year-old children can systematically predict adults’ task selection, given adults’ achievement goal (learning vs. performance) and the target child’s competence (less competent, age-matched child vs. more competent, older child).

### Methods

#### Participants.

Forty 5- and 6-year-old children (*M*_*Age*_(*SD*) = 72.33(7.51) months, Range: 60–83) in the United States were recruited via online recruitment methods (e.g., Facebook advertisements, emailing departmental participant database). Due to our novel experimental approach and within-subjects design, we were unsure about our estimated effect size, and in turn, how to power our study. As such, we based our sample size of 40 on prior work in the achievement goal literature with children that used a between subjects design (e.g., Ames, 1984; Elliott & Dweck, 1988; Harris et al., 2008).

Parents reported their children’s gender as 56% female, 42% male, and 2% preferred not to answer. The racial and ethnic makeup of participants was reported as: 57% White, 26% Asian, 11% mixed race, and 6% Black or African American, and 86% non-Hispanic or Latino, 9% Hispanic or Latino, and 5% preferred not to answer. Parental education was reported as: 3% High School or GED, 11% Associate’s degree, 46% a Bachelor’s degree, 31% a Master’s degree, and 9% Professional degree (JD, MD, PhD). Due to preregistered exclusion criteria, 16 additional participants were excluded for failing to complete the experiment (*n* = 2) or failing comprehension check questions (in the test trials; *n* = 14).

#### Materials.

Stimuli were presented on Zoom via PowerPoint (Chuey et al., 2021). The tracing stimuli were the same as in Experiment 1A and were accompanied by cartoon illustrations of adults and children with varied skin, hair, and clothing color. To depict children’s ages in our experiment, we varied children’s heights (see Figure 1).

#### Procedure.

Participants were tested virtually in a Zoom video call by an experimenter. First, participants were asked whether they knew what a tracing was and were provided with an example. As in Experiment 1A, participants saw three tracings that varied by difficulty, and were asked to identify the “easiest”, the “hardest”, and the one that was “both kind of easy and kind of hard” as comprehension checks. If participants failed any comprehension check questions, they were provided with the correct response. If participants failed all three of these comprehension questions, they were excluded from analyses based on preregistered criteria (no participants were excluded for this).

The four test trials varied the target child’s level of competence via age (age-matched vs. older) and the adult’s goal (learning vs. performance). Participants were told that they would get to see which tracings each adult chose for other children who were either the “exact same age” as the participant (less competent, age-matched child trials) or “much older” than the participant, specifically ten years of age (more competent, 10-year-old child trials). Then, participants were shown a unique adult-child pair^1^ and were told that the adult had a specific goal for the child (same wording as in Experiment 1A): to “do a perfect tracing with no mistakes” (performance goal trials) or to “learn and get better at tracing” (learning goal trials). As a comprehension check, participants were asked to repeat the adult’s goal and were corrected if they did not answer accurately. Participants were excluded if they missed comprehension checks on at least three of the four test trials (*n* = 14). For the key dependent measure, participants saw the three tracings and were asked: “Which tracing do you think this adult is going to pick for this kid—the tracing in orange, the tracing in blue, or the tracing in purple?” As in Experiment 1A, trials were blocked by child competence (less competent, age-matched child trials first, or more competent, 10-year-old child trials first) and goal order was counterbalanced within blocks.

### Preregistered Hypotheses and Analysis Plan

Based on pilot data, our preregistered hypothesis was that participants would predict an adult is more likely to choose a harder difficulty tracing for the less competent, age-matched child when they possessed a learning goal (vs. a performance goal). However, we thought that participants might find all three tracing levels too easy for a more competent, 10-year-old child and would predict that an adult would selectively choose the hardest of the three regardless of their achievement goal. Thus, we preregistered and ran an ordinal mixed-effects interaction model predicting children’s predictions of an adult’s tracing choice (1 = easy, 2 = medium, 3 = hard), as a function of the adult’s achievement goal (learning vs. performance), the child competence (less competent, age-matched vs. more competent, 10-year-old), and their interaction. This model included random slopes for achievement goal by child competence and random intercepts for participant. However, because we did not find a significant interaction but did see a main effect of achievement goal from this model (as in the adult data), we ran an ordinal mixed effects additive model (the same as in Experiment 1A) instead (same structure as above, but with no interaction term).

### Results

In line with adults’ actual choices in Experiment 1A and our preregistered hypotheses, participants predicted that adults would pick harder tracings when they held a learning (vs. performance) goal (*b* = −0.82, 95% CI [−1.47, −0.25]) and the child was more competent (vs. less competent) (*b* = 0.46, 95% CI [0.07, 0.87]) (see Figure 2). Exploratory analyses revealed that the distribution of participants’ predictions was different than chance (33% for each tracing) for both the performance and learning goal trials where the target child was more competent (*X*^2^’s(2, 40) ≥ 6.35, *p*’s ≤ 0.04) and the performance goal trial where the target child was less competent (*X*^2^(2, 40) = 6.05, *p* = 0.05), but not in the learning goal trial when the target child was less competent (*X*^2^(2, 40) = 0.8, *p* = 0.67). Exploratory binomial tests against chance of 33% (with Bonferroni *p*-value corrections, as in Experiment 1A) revealed that participants selectively chose the medium tracing for the more competent target child when the goal was to perform (*n* = 21/40, 53%, *p* = 0.03); participants did not selectively choose a tracing in the other trials (*p*’s > 0.05). Thus, although participants’ tracing predictions indeed differed depending on the achievement goal of the adult and the competence of the receiving child overall, exploratory analyses revealed that participants’ predictions did not converge on one tracing in most trials.

## EXPERIMENT 1C

To understand how children reason about adults’ achievement goals, it is important to not only look at children’s reasoning about adult actions towards other children, but also towards themselves. In Experiment 1C, we investigate children’s predictions of which tasks an adult would choose for them given specific achievement goals. As a point of comparison, and as an extension and replication of prior achievement goal work (Elliott & Dweck, 1988), we also look at how children pick tasks for themselves given different achievement goals.

### Methods

#### Participants.

Forty 5- and 6-year-old children (*M*_*Age*_(*SD*) = 72.65(7.17) months, Range: 60–83) were recruited via the same recruitment methods as in Experiment 1B. We used the same sample size we used in Experiment 1B. Parents reported their children’s gender as 60% female and 40% male. The racial and ethnic makeup of participants was reported as follows: 55% White, 17% Multiracial, 13% Asian, and 7% Black or African American, with 8% preferring not to answer, and 80% non-Hispanic or Latino and 15% Hispanic or Latino, with 5% preferring not to answer. Parental education was reported as follows: 10% Associate’s degree, 40% Bachelor’s degree, 35% Master’s degree, and 10% Professional (JD, MD, PhD) degree, with 5% preferring not to answer. Based on preregistered exclusion criteria, 6 participants were excluded for failing the majority (3/4) of comprehension questions during the test trials (*n* = 4), or experimenter error (*n* = 2).

#### Materials.

Stimuli were presented online via PowerPoint. The tracing stimuli were identical to Experiment 1B. In the trials where participants guessed what tracing adults would choose for them, a silhouetted figure was on the slide to depict said adult in addition to the tracings (see Supplementary Materials for an example). In the trials where participants were choosing tracings for themselves, they were simply presented with the tracings.

#### Procedure.

The procedure was largely similar to Experiment 1B. Participants underwent the same comprehension checks and were given four test trials. The test trials crossed achievement goals (learning vs. performance) with whether the participant was receiving a tracing from an adult (receive trials; as in Experiment 1B) or choosing a tracing for themselves (choose trials). In the receive trials, the experimenter said that two of their friends had picked out tracings for the participant after being told their age (i.e., the proxy for competence). Participants were told that the adult either had a performance goal or a learning goal for the child (using similar language as in Experiment 1B) and were asked to pick which tracing they believed that the adult chose for them. In the choice trials, participants were told to choose a tracing for themselves with either a performance goal (i.e., “make a perfect tracing with no mistakes”) or a learning goal (i.e., “help you learn and get better at tracing”) in mind. The participant role trials were blocked together (receive or choose first; order counterbalanced), and achievement goal order was counterbalanced within each block.

### Preregistered Hypotheses and Analysis Plan

We again predicted a main effect of achievement goal such that participants would expect to receive harder tracings from an adult with a learning goal (vs. a performance goal). However, based on prior research highlighting children’s over-optimism about their future performance and abilities (Leonard & Sommerville, 2025; Schneider, 1998; Zhang et al., 2025), we also predicted that participants would select harder tracings for themselves in both achievement goal trials compared to what they predict adults would select for them.

Our main analysis was an ordinal-mixed effects regression predicting participants’ tracing choices with fixed-effects and random slopes for achievement goal (learning vs. performance) and for participant role (receive vs. choose), and random intercepts for participant.

### Results

In line with our hypotheses, participants were more likely to choose harder tracings in the learning (vs. performance) goal trials (*b* = −1.69, 95% CI [−3.08, −0.62]). Contrary to our hypotheses, there was no main effect for participant role (*b* = −0.21, 95% CI [−0.97, 0.48]), meaning we did not find differences between the predictions children made for what adults would choose for them and what participants chose for themselves (see Figure 2).

Exploratory analyses revealed that the distribution of participants’ choices was different than would be expected by chance (33%) for the learning goal trials, both when predicting what adults would choose for them and when choosing for themselves (*X*^2^’s (2, 40) ≥ 6.35, *p*’s ≤ 0.04). Most participants in both the receive trial (48%) and the choose trial (50%) chose the hardest tracing when the “giver” possessed a learning goal, but these choices were not reliably above chance (*p*’s > 0.05). However, in the performance goal trials, participants’ choices were only marginally different from chance (*X*^2^’s (2, 40) ≤ 5.15, *p*’s ≥ 0.08) and participants did not selectively choose a specific tracing level in any of the trials (*p*’s > 0.05).

Taken together, these results reveal that participants were more likely to select harder tracings when they, or an adult, had a learning goal compared to a performance goal. Although children were more likely to choose the hard tracing for learning goals, exploratory analyses again revealed that their choices did not converge on a particular level for performance goals. Furthermore, contrary to our preregistered hypotheses, participants did not show any differences between their own task choices and their predictions of what tasks they would receive from adults.

## INTERIM DISCUSSION

Thus far, we found that adults are more likely to choose harder tasks for children when they hold learning over performance goals and when children are more competent (Experiment 1A). We next found that 5- and 6-year-old children predict this overall pattern when considering an adults’ task choice for third-party children (Experiment 1B), and for themselves, which parallels children’s own goal-directed choices (Experiment 1C). Collectively, these findings suggest children understand that learning and performance goals are best fulfilled by different task difficulties and should be calibrated to the task receiver’s competence.

Despite their overall similarity, we also observed discrepancies via exploratory analyses between adults’ task selection and children’s predictions. When holding a learning goal, adults showed a strong preference to choose the medium tracing for a less competent (5-year-old) child and the hard tracing for a more competent (10-year-old) child. When holding a performance goal, adults preferred to choose the easy tracing for children of both competence levels. Children, on the other hand, did not show this same selectivity when predicting adult behavior in these contexts (see Figure 2). There are two potential reasons for why children’s predictions look different than adults’ choices. First, it is possible that children have more variable representations of 5- and 10-year-olds’ competencies (which were never explicitly defined) compared to adults. Second, it may be that children believe a wider set of tasks can fulfill the same achievement goal compared to adults. In other words, even though children understand that more challenging tasks better fulfill learning vs. performance goals, they may have thought that a medium and hard difficulty task (vs. easy task) would equally satisfy a learning goal for a less competent child.

To directly test these possibilities, we made two key changes in the next experiment. First, we controlled for potential differences in participants’ beliefs about a child’s competence by explicitly defining their ability level (“Level 5” out of 11 total levels) instead of using age as a proxy. Second, we introduced participants to a task space that is more clearly defined and has a broader range (e.g., this is a “Level 4” task in a space of tasks from Levels 0–10). In addition to clarifying results from Experiment 1, this new task design also enabled us to test whether adults (Experiment 2A) and children (Experiment 2B) believe that learning goals are best achieved by selecting any challenging task *or*, instead, a task just one level beyond a child’s current ability. This latter idea aligns with classic developmental theories on optimal learning (van der Veer & Valsiner, 1991; Vygotsky, 1978; Wass & Golding, 2014).

## EXPERIMENT 2A

In Experiment 2A, we explore whether adults not only think harder tasks better fulfill learning (vs. performance) goals, but also whether they think tasks just one level harder than a child’s current competency best fulfill this goal. Participants were introduced to children whose competence was explicitly stated (e.g., this child can read up to “Level 5” books) and asked to choose a book, from Level 0 to Level 10 for each child given a goal for the child to learn or perform.

Aiming to replicate findings from Experiment 1A, we first tested whether adults chose harder tasks when they had learning (vs. performance) goals and when the receiving child was more competent. To look at the specificity of adult’s choices, we also ran exploratory analyses to see whether adults with a learning goal would selectively choose a task just a level above a child’s current competency. This behavior would be in line with classic theory on the zone of proximal development (Vygotsky, 1978), which posits that the most effective teaching is achieved through challenging children with tasks just above their current competencies. In contrast to learning goals, many tasks can equally fulfill performance goals. To achieve a successful outcome, giving a child a task just below or any level below the child’s current competency will suffice.

### Methods

#### Participants.

Fifty adults (*M*_*Age*_(*SD*) = 44.28(12.72) years, Range: 24–73) were recruited via Amazon Mechanical Turk. Participants self-reported their gender as 68% male, 30% female, and 2% non-binary. The racial and ethnic makeup of participants was reported as: 84% White, 8% Black or African American, 4% Asian, 2% Multiracial, and 92% non-Hispanic or Latino, 6% Hispanic or Latino, and 2% preferred not to answer. An additional 22 participants were collected but excluded for failing either comprehension checks, or an attention check based on preregistered criteria.

#### Materials.

An online survey was created and administered using the Qualtrics platform. A “Reading Level tracker” depicted 11 reading levels, from Level 0 (easiest books) to Level 10 (hardest books). A solid arrow was used to mark the Reading Level of an individual child. Six unique silhouettes of children were used across test trials (see Supplementary Materials Figure S3).

#### Procedure.

Participants were told that they would be choosing books for children to read that ranged in difficulty from Level 0 (easiest books) to Level 10 (hardest books). Participants who failed to correctly identify the easiest and hardest level books (two comprehension check questions) were excluded (*n* = 21).

In the test trials, participants were told the reading level of each child that they were choosing books for (e.g., “Now you are going to pick a Reading Level for a kid that is a ‘Level [3/5/7]’. That means that this kid can read the books from all the levels up to and including Level ‘[3/5/7]’”.). As in Experiment 1A, participants were asked to hold either a learning goal (i.e., “help the kid learn and get better at reading”) or a performance goal (i.e., “help the kid earn a sticker^2^ by reading perfectly with no mistakes”) when choosing a book for a child. Each participant underwent six test trials (order randomized) crossing their held achievement goal (learning vs. performance) with the target child’s competence (Reading Level 3, 5, or 7).

### Preregistered Hypotheses and Analysis Plan

We aimed to replicate findings from Experiment 1, showing that adults choose harder tracings when they have a learning goal (vs. performance goal) and when the target child has higher competence. To this end, we ran a linear mixed-effects regression predicting participants’ Reading Level choices (0–10) with fixed effects and random slopes for achievement goal (learning vs. performance) and child competence (Reading Level 3, Level 5, vs. Level 7), and random intercepts for participant. As in Experiments 1A–C, we also ran separate models within learning and performance goal trials, as well as all three child competence trials separately (see Supplementary Materials for details).

### Results

**Figure 3.**
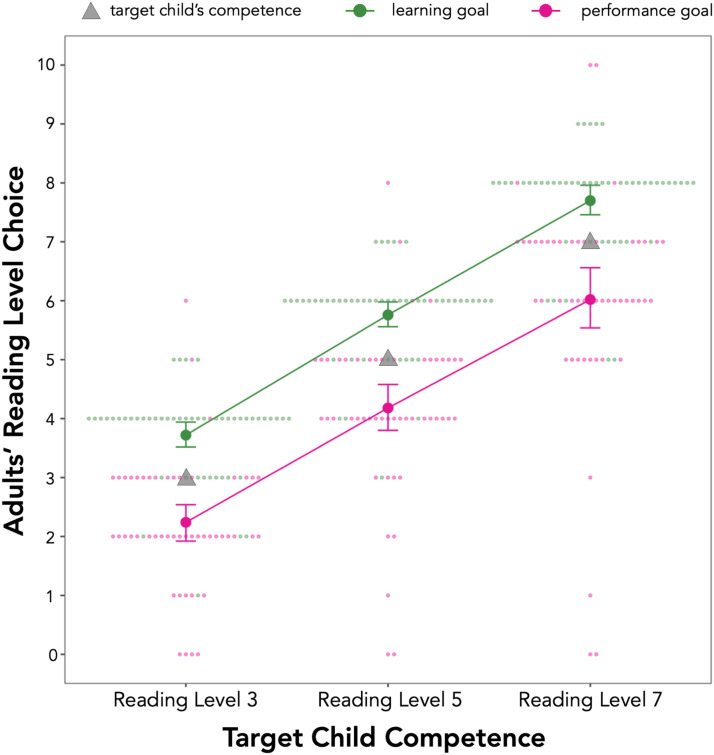
**Results from Experiment 2A.** Adults selectively chose a book just one level above a child’s mastery level when they had a learning goal and just one level below when they had a performance goal. The *X*-axis shows different child competence trials (Reading Level 3, 5, or 7 from a range of 0 to 10) and the *Y*-axis shows participants’ reading level selections (from the easiest Reading Level 0 to the hardest Reading Level 10) given either a learning goal (in green) or a performance goal (in pink). Large dots represent participants’ average response, small dots represent individual responses, and gray triangles represent children’s competence level within each trial. Error bars represent 95% bootstrapped CIs.

In line with results from Experiment 1A and our preregistered hypotheses, participants were more likely to choose higher Reading Levels when they possessed a learning goal than a performance goal (*b* = 1.58, *p* < 0.001) and when the target child was more competent (Reading Level 5 vs. Reading Level 3: *b* = 1.99, *p* < 0.001; Reading Level 7 vs. Reading Level 3: *b* = 3.88, *p* < 0.001) (see Figure 3).

We next ran exploratory analyses looking at the specificity of participants’ task choice selections given children’s competence and their achievement goal. To create a measure of how much adults’ selections over or undershot the child’s competence, we subtracted the target child’s reading level from each participant’s book level choice. We used this measure as the dependent variable in our analyses. For learning goal trials, participants on average chose a book that was 0.73 levels above (95% CI: [0.60, 0.86]) the child’s reading level. The modal response was to choose one level up for children at a Reading Level 3 (66% chose a level 4 book, *p* < 0.001, binomial test against chance = 9% or 1 out of 11), Reading Level 5 (66% chose a level 6 book, *p* < 0.001, binomial test against chance = 9%), and Reading Level 7 (64% chose a level 8 book, *p* < 0.001, binomial test against chance = 9%). For performance goals, on average, participants chose a book that was 0.85 levels (95% CI: [0.61, 1.09]) below the child’s reading level. The modal response was to choose a book one level below the child’s reading level for a child at a Reading Level 3 (44% chose a level 2 book, *p* < 0.001, binomial test against chance = 9%) and a Reading Level 5 (40% chose a level 4 book, *p* < 0.001, binomial test against chance = 9%). However, participants’ choices were split between a level 6 book and a level 7 book for a child at a Reading Level 7 (34% chose a book at each level, *p*’s < 0.001, binomial test against chance = 9%). These exploratory findings suggest that adults with a learning goal tend to choose a book one level up from a child’s competence and adults with a performance goal tend to choose a book one level down from a child’s competence (see Figure 3).

Taken together, these results replicate and extend our findings from Experiment 1A: When asked to choose a task for a child, adults consider both their achievement goal and the child’s competence. Moreover, exploratory analyses suggest that adults specifically tailor their task selection to children’s competence, choosing to give tasks just a bit out of reach to children when they have a learning goal and tasks just a bit easier when they have a performance goal.

## EXPERIMENT 2B

In Experiment 2B, we test whether children also have specific intuitions about which level of task difficulty best fulfills each achievement goal. As in Experiment 2A, we introduced participants to children who wanted to read books. To reduce task demands and to closely match the designs of Experiments 1B and 1C, we opted to use a modified version of Experiment 2A that allowed children to choose between two options, rather than to pick from 11 options. Additionally, we increased our age range to include 7- to 8-year-old children so that we could explore any potential age effects in children’s predictions. Participants were introduced to two teachers who had the same goal (learning or performance) but made different task choices for children who were a Reading Level 5. Our critical question was which teacher participants thought best helped the student achieve a learning or performance goal.

### Methods

#### Participants.

Eighty 5- to 8-year-old children (*M*_*Age*_(*SD*) = 83.98(13.48) months, Range: 60–106) were recruited via online recruitment methods. We used the same sample size as Experiment 1B and 1C for our 5- to 6-year-old participants. However, because we extended the age range to now include 7- and 8-year-old children, we increased the sample to include an even amount within each age bin in years (*n* = 20). Parents reported their children’s gender as 60% female and 40% male. The racial and ethnic makeup of participants was reported as follows: 64% White, 14% Asian, 11% Multiracial, 3% Black or African American, and 3% American Indian or Alaskan with 6% preferring not to answer, and 79% non-Hispanic or Latino and 14% Hispanic or Latino, with 7% preferring not to answer. Parental education was reported as follows: 6% High School or GED, 3% Associate’s degree, 33% Bachelor’s degree, 35% Master’s degree, and 20% Professional (JD, MD, PhD) degree, with 3% preferring not to answer. Based on preregistered exclusion criteria, 23 participants were excluded for failing comprehension questions (in either the warm-up or test trials; *n* = 15), failing to complete the experiment (*n* = 4), or experimenter or technical error (*n* = 4).

#### Materials.

Stimuli were presented online via PowerPoint. The “Reading Level tracker” was the same as in Experiment 2A. The same silhouettes of children (used in Experiment 2A) and additional teacher silhouettes were also used in this experiment.

#### Procedure.

Participants were tested virtually in a Zoom video call by an experimenter. Children were introduced to a classroom where teachers pick out books for students to read. These students have “Reading Levels” ranging from Level 0 to Level 10, that are marked on a “Reading Level tracker” (the same used in Experiment 2A). Children then underwent a series of comprehension check questions in order to ensure they understood how task difficulty and child competence mapped onto this Reading Level scale (see Supplementary Materials for details).

Children were told that some teachers wanted “to help kids to learn and get better at reading” (i.e., a learning goal), while some teachers wanted “to help kids earn some stickers by reading perfectly with no mistakes” (i.e., a performance goal). Then, participants underwent four test trials (within-subjects) where they were introduced to two teachers with the same achievement goal (either a learning or performance goal) but chose different books for a student at a Reading Level 5 (note that this reading level was kept constant in this paradigm). As a comprehension check, children were asked to repeat the teachers’ achievement goals in the first two trials. If children failed to correctly state the teacher’s goal on either trial, after one correction, their data was excluded (*n* = 15).

To confirm that children thought that learning goals were best satisfied by choosing harder tasks and performance goals were best satisfied by choosing easier tasks, children were shown two teachers with the same achievement goal (learning or performance) for a child at a Reading Level 5. The key difference between the teachers was that one chose a Level 6 book, and one chose a Level 4 book. For example, when both teachers had a learning goal, participants were shown each teacher’s task choice and asked our critical question: “Which teacher would best help the student learn and get better at reading?” The performance goal trial was identical in format except for the stated achievement goal and the wording of the test question: “Which teacher would best help the student earn a sticker?”. These two trials were always presented first to participants (order counterbalanced).

The next two trials tested whether children have predictions about how much harder or easier the task should be for each goal. In the learning goal trial, one teacher chose a Level 6 (i.e., indicating a preference for one level above the student’s level) and the other chose a Level 8 (i.e., indicating a preference for a much harder task). In the performance goal trial, one teacher chose a Level 4 (i.e., indicating a preference for one level below the student’s level) and the other chose a Level 2 (i.e., indicating a preference for a much easier task). These two trials were always presented in a second block (order counterbalanced)^3^.

### Preregistered Hypotheses and Analysis Plan

For the first block of trials, our main hypothesis was that children would say the teacher who chose a harder Reading Level (Level 6) would best satisfy a learning goal and that a teacher who chose an easier Reading Level (Level 4) would best satisfy a performance goal. We fit a mixed-effects logistic regression predicting children’s teacher choice with fixed effects for achievement goal (learning vs. performance) and participant age (continuous, in months), a random slope for achievement goal, and random intercepts for participant.

For the second block of trials, our hypothesis was that children would be sensitive to which levels of difficult tasks best fulfill learning goals, as reflected in adults’ own choices in Experiment 2A. Specifically, we predicted that children would think that the teacher with a learning goal who chose a Level 6 book for a Level 5 reader would better fulfill their goal than a teacher with a learning goal who chose a Level 8 book for the same child. We remained agnostic as to which teacher (one who chose Level 2 or Level 4) children would prefer when the teacher held a performance goal as both choices may satisfy the goal. To test these predictions, we ran binomial tests comparing participants’ teacher choices to chance (50%) within each trial.

### Results

**Figure 4.**
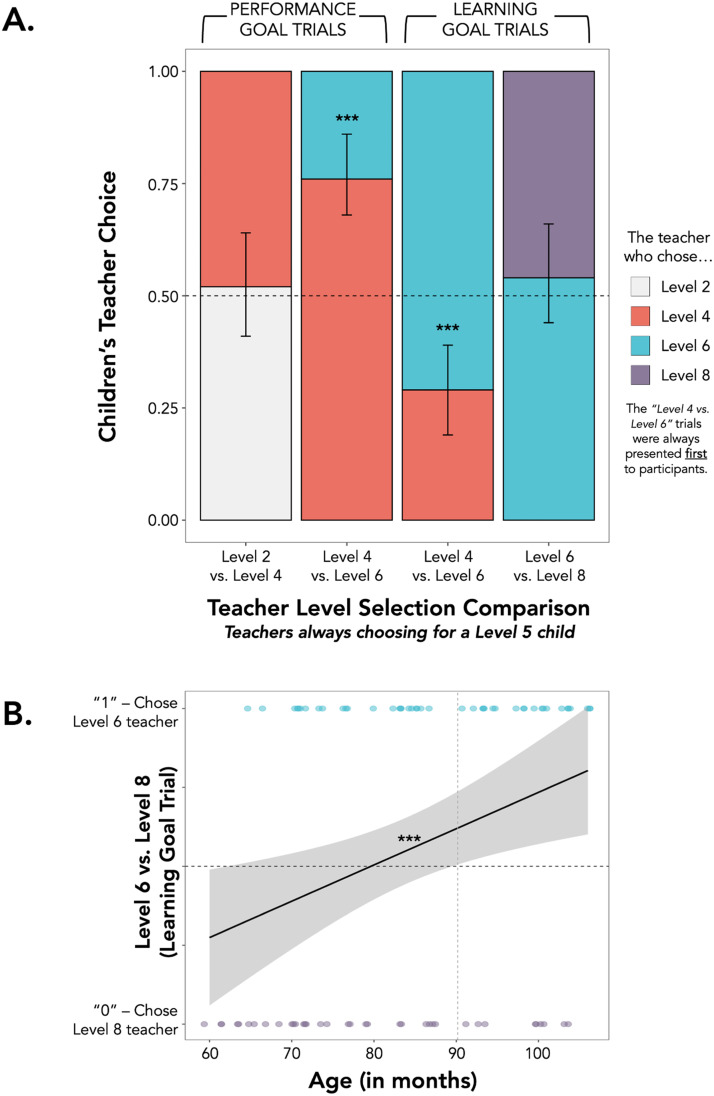
**Results from Experiment 2B.** (A) Children were tasked with choosing which of two teachers did a better job fulfilling their goal—for the child to perform (left bars) or learn (right bars)—with their book choice (*X*-axis) for a “Level 5” reader. The *X*-axis shows teachers’ choices on each test trial and the *Y*-axis shows the proportion of participants’ teacher choice within each trial. Error bars represent 95% bootstrapped CIs. (***p < 0.001). Results show that children think that learning goals are best fulfilled by giving children harder tasks and that performance goals are best fulfilled by giving children easier tasks (“Level 4 vs. 6” trials in the middle; always presented first), but they do not have strong beliefs about how much harder or easier the tasks should be to fulfill each goal (trials on far right and far left; presented after). (B) Participants’ choices in the “Level 6 vs. Level 8” learning goal trial as a function of age. The *X*-axis shows participant age in months, and the *Y*-axis depicts a participant’s choice of the teacher who chose a Level 6 book (coded as 1) or the teacher who chose a Level 8 book (coded as 0). Shaded areas represent 95% CIs. (***p < 0.001). The vertical, gray dashed line marks the age (90.17 months) at which participants begin to reliably select the Level 6 teacher above chance (0.50), marked with the black dashed line. In other words, by 90.17 months, children start to believe that giving tasks just a level harder (vs. a lot harder) than one’s current ability best fulfills a learning goal.

As predicted, children expected that learning goals are best fulfilled by giving children harder tasks and that performance goals are best fulfilled by giving children easier tasks. Specifically, children chose the Level 6 book teacher more than the Level 4 book teacher when the teachers possessed learning goals versus performance goals (*b* = −2.89, *p* < 0.001). There was no main effect of participant age (*b* = 0.02, *p* = 0.36). When both teachers possessed a learning goal, the majority of children (71%) said the teacher who chose a Level 6 book better satisfied the goal (*p* < 0.001, binomial test against chance 50%). However, when both teachers possessed a performance goal, the majority of children (76%) said the teacher who chose a Level 4 book better satisfied the goal (*p* < 0.001, binomial test against chance 50%; see Figure 4).

Next, we examined whether participants had predictions about which levels of harder or easier tasks best fulfilled each achievement goal. When both teachers possessed a performance goal, participants did not show a preference between the teacher who chose a Level 2 book and the teacher who chose a Level 4 book (Level 2 vs. Level 4: 53% vs. 47%, *p* = 0.74; binomial test against chance, 50%). Thus, unlike adults, child participants may believe that *any* task that is easier than a child’s current level will satisfy a performance goal. Additionally, contrary to our preregistered predictions, when both teachers possessed a learning goal, participants did not show a preference between the teacher who chose a Level 6 book over a teacher who chose a Level 8 book (Level 6 vs. Level 8: 54% vs. 46%, *p* = 0.43, binomial test against chance, 50%). Thus, unlike adults, participants did not believe that choosing a task just a level up from (vs. a lot harder than) a child’s current competency level will best help them learn.

We next ran exploratory analyses to examine whether, with age, children possessed adult-like intuitions on which tasks best fulfill learning and performance goals. A logistic regression predicting participants’ teacher choices in the Level 2 vs. 4 performance goal trial with a fixed effect of participant age found no main effect of age (*b* = −0.01, *p* = 0.38). Thus, across age, children were at chance when deciding between a teacher who chooses a Level 2 and a teacher who chooses a Level 4 would better satisfy a performance goal. However, a logistic regression predicting participants’ teacher choices in the Level 6 vs. 8 learning goal trial with a fixed effect of participant age revealed a positive effect of participant age (*b* = 0.01, *p* < 0.01). In other words, as children get older, they are more likely to say that a teacher who chooses a Level 6 (vs. 8) book will best satisfy a learning goal for the Level 5 student. To determine the exact age at which children reliably choose the Level 6 teacher, we computed the 95% CIs for the predicted log-odds of choosing the Level 6 teacher from age 5 to 8 (continuous) and found the point at which the lower bound of the CI crossed 50% (chance level). This exploratory analysis revealed that at around 7.5 years old (90.17 months), children were more likely to say that the Level 6 teacher better satisfied the learning goal, compared to the Level 8 teacher (see Figure 4B).

## GENERAL DISCUSSION

Decades of research have demonstrated that achievement goals shape children’s learning, motivation, and academic success (Elliott & Dweck, 1988; Grant & Dweck, 2003; Heyman & Dweck, 1992). While much of this work has focused on the effects of children’s own goals, less is known about how children reason about the achievement goals of others, particularly the adults who often guide their early learning. Here, we addressed this gap by examining whether children understand how adults’ achievement goals translate into specific child-directed actions.

We first verified that adults choose more difficult tasks for children when they possess learning (vs. performance) goals and when a child is more competent. Specifically, when adults want children to learn, they select tasks slightly more challenging than the child’s current skill level, and when they want children to perform, they choose tasks that are slightly easier than what the child has already mastered. Second, we found that 5- to 6-year-old children broadly predict this pattern when reasoning about tasks given to other children and to themselves. In both adults and children, these beliefs emerged in the absence of direct pedagogical training, suggesting that people hold intuitive expectations about how learning should occur. However, contrary to our predictions, children were agnostic as to how much harder or easier these tasks should be given a child’s skill. Exploratory analyses revealed that, with age, 5- to 8-year-old children’s predictions become more aligned with adults’ actual choices, showing an increasing understanding that learning goals are best fulfilled by giving children incrementally harder tasks.

Our findings provide two key contributions to the achievement goal literature. First, our work demonstrates that children can explicitly reason about how adults’ achievement goals are reflected in their task choices. As such, our work is the first to show that children’s rich, causal reasoning capacities (e.g., Goddu & Gopnik, 2024; Gopnik & Schulz, 2007; Walker & Gopnik, 2014) may extend to the abstract, achievement goal domain: Children are able to reason about how adults’ internal goal states (for the child to learn or perform) map onto observable actions (task selection). Second, our results show that both adults and children make systematic predictions about achievement goals rather than relying on simple heuristics (e.g., learning goal = harder task choice). Even 5- to 6-year-old children understand that a 10-year-old should receive harder tasks than a 5-year-old when trying to learn. However, with age, children show adult-like reasoning and appreciate not only the direction of difficulty needed (harder or easier), but also how much harder the task ought to be to fulfill a learning goal given someone’s skill. Given the exploratory nature of this age-related change result, future work is needed to ensure its robustness.

Why do older children, but not younger children, understand that incrementally more difficult task choices would best help someone learn? One possibility is that younger children hold different beliefs about the learning process. Prior work on metacognition suggests that children younger than 6 might not fully understand that the learning process necessitates practice and time (Sobel & Letourneau, 2015; Zhang et al., 2025) and thus may think that any significantly harder task can help someone learn. Another non-mutually exclusive possibility from work on ecological, adaptive learning suggests that young children may simply not think strategically about sampling. Young children tend to engage in broad, exploratory information-sampling, whereas older children increasingly specialize their sampling and exploit options that appear most rewarding or informative (Giron et al., 2023; Nussenbaum et al., 2023; Ruggeri, 2022). As such, young children may not intuit that effective learning involves targeted exploration calibrated to one’s current skill level. For both of these possibilities, broader cognitive development (e.g., metacognitive or executive function) and/or experiences (e.g., formal schooling) may fuel children’s conceptual understanding of learning. For example, in formal schooling, difficulty and skill levels are often made highly salient through practices like reading levels or ability grouping, which may help children realize that learning is best supported by incremental challenge. Future work is needed to fully understand what beliefs, cognitive capacities, and experiences enable individuals to intuit that slightly more challenging tasks are most effective for learning.

Our work showing that older children and adults anchor their achievement goal related task choice based on a child’s competency has a number of important implications. In the real world, children’s competencies are not always apparent and usually must be inferred, which leaves room for bias. For example, adults’ stereotyped beliefs about socioeconomic status (SES) and gender result in differential treatment that impacts real-world outcomes and perpetuates disparities in the workforce and classroom (Brummelman & Sedikides, 2023; Carlana, 2019; Cimpian et al., 2020; Doyle et al., 2023; Maaz et al., 2008; Newall et al., 2018; Rosenthal & Jacobson, 1968; Rubovits & Maehr, 1973; Tenenbaum & Ruck, 2007). As such, adults’ achievement-oriented guidance may sometimes be suboptimal: For example, a teacher who assumes that girls have lower math abilities than boys may assign girls tasks that are too easy, despite intending to help girls learn. Furthermore, the overall difficulty of accurately tracking children’s quickly growing capabilities may make achievement-guided actions tailored to children’s abilities genuinely challenging (Shachnai et al., 2025). Future work should address the ways in which adults can better learn about children’s competence, such that they can best support children’s learning and achievement.

Given that children can predict actions from achievement goals, a natural next question is whether they can infer achievement goals from actions. If so, children may then learn about adults’ values simply from their actions. For example, a child might learn that an adult who consistently gives them hard tasks wants them to learn (vs. perform) and thus opt to engage in more learning opportunities in front of them (e.g., Good & Shaw, 2021). In the classroom, children may pick up on teachers’ differential goals for learning vs. performance across contexts given their task choice for them. For example, a student may infer that a teacher who gives them hard math problems but easy music pieces to play wants them to learn in math class but perform in the orchestra. This type of inference can support children’s understanding of what behavior is expected of them across contexts, as well as potentially inform their representations of themselves.

A related question is whether children can infer competence given others’ goals and actions. Imagine the following scenario: A tutor gives the same activity to Sally and to Anne. However, for Sally, the tutor emphasizes that the goal of the activity is to learn, and, for Anne, the goal is to do the activity perfectly^4^. Based on our present findings, young children might infer that Anne is more competent than Sally since Anne can presumably do the activity perfectly, but Sally is still learning to do it well. These inferences would be consistent with prior work showing that children form early ability beliefs from cues in their social environment like praise (Gunderson et al., 2013, 2018), others’ emotional expressions and nonverbal behaviors (Asaba et al., 2025; Brey & Shutts, 2018), and even wealth (Shutts et al., 2016). As such, educators who have unique achievement goals for children but provide identical learning materials may inadvertently allow children to make conclusions about their competence via these peer-to-peer comparisons. Future work should explore how children draw inferences about their own and others’ competence given adults’ achievement goal-directed behaviors.

Across our experiments, children and adults were asked to select only a single task (or level of difficulty) as a means of satisfying a particular achievement goal. This design reflects many daily, discrete choices adults make for children: parents choosing a book, teachers assigning an activity, or coaches selecting a drill. However, such discrete choices are often embedded within sequences of tasks intended to foster learning. For example, when a child masters a task, educators typically introduce a more difficult one if their goal is to promote growth (e.g., Ornstein, 1997). This raises the question of how children reason about and represent achievement goals over time. Might children infer different goals when adults select progressively harder tasks versus consistently similar ones? Given prior work showing that young children are sensitive to performance trajectories (Leonard et al., 2023; Zhang et al., 2025) and can adaptively structure their own learning curricula (Bass et al., 2023; Dahmani et al., 2025; Serko et al., 2025), children may also be attuned to how others’ sequencing of tasks maps onto their achievement goals. Future work should directly investigate both how adults structure sequences of tasks given different achievement goals and how children interpret these goal-directed patterns of behavior.

Our work has a number of limitations. We only tested children’s predictions within the classic dichotomy of learning and performance goals (Ames, 1992; Ames & Archer, 1988; Elliott & Dweck, 1988). We specifically described a performance goal focused solely on non-normative avoidance (Carver, 2006; Strauman & Wilson, 2010) and a learning goal focused solely on non-normative approach (Guo et al., 2023). Future work is necessary to see whether children accurately predict adult’s actions across the many types of achievement goals that exist across both different points of comparison (e.g., comparisons to others, the self, or a task-based standards) (see the 3 × 2 model of achievement goals; Elliot et al., 2011) and approach and avoid contexts (e.g., desiring extrinsic validation for successful task performance; avoiding tasks that will not help them learn) (see Dweck & Leggett, 1988; Elliot & Harackiewicz, 1996; Elliot & McGregor, 2001; Midgley et al., 2001). For example, it seems plausible that if an adult wanted their child to show off to judges by performing better than other children (a normative, performance–approach goal) they might reasonably pick a task that was at the current level of the child’s abilities. If this adult similarly was not concerned about the child making mistakes, they may even pick a task just above the child’s current level of ability (in line with work highlighting how performance-approach goal orientations can sometimes be linked to positive child outcomes; Elliot & Moller, 2003). As such, we do not take our results as evidence for the broader claim that children always assume that performance goals lead adults to select easier tasks than learning goals. Future work could also explore contexts where adults simultaneously want their child to learn and succeed. This type of joint goal may encourage children to choose a task of desirable difficulty, or rather default to prioritizing one goal over the other.

Additionally, our paradigm provided children with adults’ explicit achievement goals, but this is often not the case in the real world. It remains an open question whether and how children might infer an adult’s achievement goals from just their actions. Furthermore, several of the findings we report are exploratory rather than confirmatory, so future work is needed to determine their robustness. Finally, our sample comes from predominantly White, highly educated backgrounds, and was collected from a WEIRD country (Henrich et al., 2010), which limits the generalizability of our results to broader populations.

## CONCLUSION

If you pull back the curtain behind children’s achievement, there is often a group of dedicated adults, like parents and teachers, guiding their development through the selection of certain tasks and activities. Here, we show that children have a deeper understanding of how adults’ achievement goals give rise to such actions. Specifically, children predict that an adult who wants them to learn will pick a harder task for them than an adult who wants them to perform. As children age, they begin to intuit, as adults do, that learning goals are best fulfilled by giving children tasks just a bit harder than their current competency. This work suggests that children have a rich mental model of how best to fulfill achievement goals—the very same goals that will shape their own relationship with their learning, motivation, and success in years to come.

## ACKNOWLEDGMENTS

We thank all members of the Leonard Learning Lab, Julian Jara-Ettinger, and Frank Keil for helpful discussions about this work. We also thank Melissa Santos and Natalie Massetti for their help with participant recruitment, online testing administration, and data collection. We are extremely grateful to Lizbeth Lozano, Lauren Okine, Christina Norberg, Paloma Casanova, and Bethel Asomaning for their help with data collection and coding.

## FUNDING INFORMATION

This work was funded by a Jacobs Foundation research grant to J. A. L.

## AUTHOR CONTRIBUTIONS

B.A.C.: Conceptualization; Data curation; Formal analysis; Investigation; Methodology; Project administration; Validation; Visualization; Writing – original draft; Writing – review & editing. M.A.: Methodology; Validation; Visualization; Writing – original draft; Writing – review & editing. J.A.L.: Conceptualization; Funding acquisition; Methodology; Resources; Supervision; Writing – original draft; Writing – review & editing.

## DATA AVAILABILITY STATEMENT

All experiment scripts, materials, data, and code can be found on OSF: https://tinyurl.com/zmjnf37r.

## Notes

^1^ We purposefully did not specify the adult’s relationship with the child (e.g., parent, caregiver, teacher), to avoid any associations children have about specific adult roles and the goals adults in those roles typically hold (e.g., that teachers typically want children to learn).^2^ As in our first experiment, we limited our description of a performance goal to focus on ability and performance-avoidance. We added more context (i.e., earn a sticker) to our performance goal description to emphasize further that the focus of the goal was solely on performance and not any alternative social pressures, like impressing others.^3^ Thus, even if children choose a Level 4 teacher on the initial learning goal trial, they were still asked to choose between a Level 6 and 8 teacher in the second block learning goal trial.^4^ Although achievement goal climate is often theorized as a classroom effect, educators and caretakers also have unique goals for specific children (Gonida & Cortina, 2014; Mageau et al., 2016; Robinson, 2023).

## Supplementary Material



## References

[bib1] Ablard, K. E., & Parker, W. D. (1997). Parents’ achievement goals and perfectionism in their academically talented children. Journal of Youth and Adolescence, 26(6), 651–667. 10.1023/A:1022392524554

[bib2] Ames, C. (1984). Achievement attributions and self-instructions under competitive and individualistic goal structures. Journal of Educational Psychology, 76(3), 478–487. 10.1037/0022-0663.76.3.478

[bib3] Ames, C. (1992). Classrooms: Goals, structures, and student motivation. Journal of Educational Psychology, 84(3), 261–271. 10.1037/0022-0663.84.3.261

[bib4] Ames, C., & Archer, J. (1988). Achievement goals in the classroom: Students’ learning strategies and motivation processes. Journal of Educational Psychology, 80(3), 260–267. 10.1037/0022-0663.80.3.260

[bib5] Asaba, M., Wu, Y., Carrillo, B., & Gweon, H. (2025). When success is surprising: Children’s ability to use surprise to infer competence. Open Mind: Discoveries in Cognitive Science, 9, 825–843. 10.1162/opmi.a.2, 40697897 PMC12283150

[bib6] Baer, C., & Odic, D. (2022). Mini managers: Children strategically divide cognitive labor among collaborators, but with a self-serving bias. Child Development, 93(2), 437–450. 10.1111/cdev.13692, 34664258

[bib7] Bardach, L., Oczlon, S., Pietschnig, J., & Lüftenegger, M. (2020). Has achievement goal theory been right? A meta-analysis of the relation between goal structures and personal achievement goals. Journal of Educational Psychology, 112(6), 1197–1220. 10.1037/edu0000419

[bib8] Barr, D. J., Levy, R., Scheepers, C., & Tily, H. J. (2013). Random effects structure for confirmatory hypothesis testing: Keep it maximal. Journal of Memory and Language, 68(3), 255–278. 10.1016/j.jml.2012.11.001, 24403724 PMC3881361

[bib9] Bass, I., Mahaffey, E., & Bonawitz, E. (2023). Children use teachers’ beliefs about their abilities to calibrate explore–exploit decisions. Topics in Cognitive Science. 10.1111/tops.12714, 38033200

[bib10] Brey, E., & Shutts, K. (2018). Children use nonverbal cues from an adult to evaluate peers. Journal of Cognition and Development, 19(2), 121–136. 10.1080/15248372.2018.1449749, 30443199 PMC6234014

[bib11] Bridgers, S., Jara-Ettinger, J., & Gweon, H. (2020). Young children consider the expected utility of others’ learning to decide what to teach. Nature Human Behaviour, 4(2), 144–152. 10.1038/s41562-019-0748-6, 31611659

[bib12] Brummelman, E., & Sedikides, C. (2023). Unequal selves in the classroom: Nature, origins, and consequences of socioeconomic disparities in children’s self-views. Developmental Psychology, 59(11), 1962–1987. 10.1037/dev0001599, 37650811

[bib13] Carlana, M. (2019). Implicit stereotypes: Evidence from teachers’ gender bias. Quarterly Journal of Economics, 134(3), 1163–1224. 10.1093/qje/qjz008

[bib14] Carver, C. S. (2006). Approach, avoidance, and the self-regulation of affect and action. Motivation and Emotion, 30(2), 105–110. 10.1007/s11031-006-9044-7

[bib60] Chu, J., Rule, J., Goddu, M., Pinter, V., Reagan, E. R., Bonawitz, E., Gopnik, A., & Ullman, T. D. (2025). Fun isn’t easy: Children selectively manipulate task difficulty when “playing for fun” versus “playing to win”. Developmental Psychology. 10.1037/dev0002108, 41379658

[bib15] Chuey, A., Asaba, M., Bridgers, S., Carrillo, B., Dietz, G., Garcia, T., Leonard, J. A., Liu, S., Merrick, M., Radwan, S., Stegall, J., Velez, N., Woo, B., Wu, Y., Zhou, X. J., Frank, M. C., & Gweon, H. (2021). Moderated online data-collection for developmental research: Methods and replications. Frontiers in Psychology, 12, 734398. 10.3389/fpsyg.2021.734398, 34803813 PMC8595939

[bib16] Cimpian, J. R., Kim, T. H., & McDermott, Z. T. (2020). Understanding persistent gender gaps in STEM. Science, 368(6497), 1317–1319. 10.1126/science.aba7377, 32554586

[bib17] Dahmani, A., Yiu, E., & Gopnik, A. (2025). Children spontaneously design curricula to tackle challenging tasks. In D. Barner, N. R. Bramley, A. Ruggeri, & C. M. Walker (Eds.), Proceedings of the 47th Annual Conference of the Cognitive Science Society (pp. 5859–5865). Cognitive Science Society.

[bib18] Doyle, L., Easterbrook, M. J., & Harris, P. R. (2023). Roles of socioeconomic status, ethnicity and teacher beliefs in academic grading. British Journal of Educational Psychology, 93(1), 91–112. 10.1111/bjep.12541, 35998351 PMC10087759

[bib19] Dweck, C. S., & Leggett, E. L. (1988). A social-cognitive approach to motivation and personality. Psychological Review, 95(2), 256–273. 10.1037/0033-295X.95.2.256

[bib20] Dweck, C. S., & Yeager, D. S. (2019). Mindsets: A view from two eras. Perspectives on Psychological Science, 14(3), 481–496. 10.1177/1745691618804166, 30707853 PMC6594552

[bib21] Elliot, A. J., & Harackiewicz, J. M. (1996). Approach and avoidance achievement goals and intrinsic motivation: A mediational analysis. Journal of Personality and Social Psychology, 70(3), 461–475. 10.1037/0022-3514.70.3.461 8014838

[bib22] Elliot, A. J., & Hulleman, C. S. (2017). Achievement goals. In A. J. Elliot, C. S. Dweck, & D. S. Yeager (Eds.), Handbook of competence and motivation: Theory and application (2nd ed., pp. 43–60). The Guilford Press.

[bib23] Elliot, A. J., & McGregor, H. A. (2001). A 2 × 2 achievement goal framework. Journal of Personality and Social Psychology, 80(3), 501–519. 10.1037/0022-3514.80.3.501, 11300582

[bib24] Elliot, A. J., & Moller, A. C. (2003). Performance-approach goals: Good or bad forms of regulation? International Journal of Educational Research, 39(4–5), 339–356. 10.1016/j.ijer.2004.06.003

[bib25] Elliot, A. J., Murayama, K., & Pekrun, R. (2011). A 3 × 2 achievement goal model. Journal of Educational Psychology, 103(3), 632–648. 10.1037/a0023952

[bib26] Elliott, E. S., & Dweck, C. S. (1988). Goals: An approach to motivation and achievement. Journal of Personality and Social Psychology, 54(1), 5–12. 10.1037/0022-3514.54.1.5, 3346808

[bib27] Giron, A. P., Ciranka, S., Schulz, E., van den Bos, W., Ruggeri, A., Meder, B., & Wu, C. M. (2023). Developmental changes in exploration resemble stochastic optimization. Nature Human Behaviour, 7(11), 1955–1967. 10.1038/s41562-023-01662-1, 37591981 PMC10663152

[bib28] Goddu, M. K., & Gopnik, A. (2024). The development of human causal learning and reasoning. Nature Reviews Psychology, 3(5), 319–339. 10.1038/s44159-024-00300-5

[bib29] Gonida, E. N., & Cortina, K. S. (2014). Parental involvement in homework: Relations with parent and student achievement-related motivational beliefs and achievement. British Journal of Educational Psychology, 84(3), 376–396. 10.1111/bjep.12039, 24905081

[bib30] Gonida, E. N., Voulala, K., & Kiosseoglou, G. (2009). Students’ achievement goal orientations and their behavioral and emotional engagement: Co-examining the role of perceived school goal structures and parent goals during adolescence. Learning and Individual Differences, 19(1), 53–60. 10.1016/j.lindif.2008.04.002

[bib31] Good, K., & Shaw, A. (2021). Achieving a good impression: Reputation management and performance goals. Wiley Interdisciplinary Reviews: Cognitive Science, 12(4), e1552. 10.1002/wcs.1552, 33426784

[bib32] Gopnik, A., & Schulz, L. (Eds.). (2007). Causal learning: Psychology, philosophy, and computation. Oxford University Press. 10.1093/acprof:oso/9780195176803.001.0001

[bib33] Grant, H., & Dweck, C. S. (2003). Clarifying achievement goals and their impact. Journal of Personality and Social Psychology, 85(3), 541–553. 10.1037/0022-3514.85.3.541, 14498789

[bib34] Gunderson, E. A., Gripshover, S. J., Romero, C., Dweck, C. S., Goldin-Meadow, S., & Levine, S. C. (2013). Parent praise to 1- to 3-year-olds predicts children’s motivational frameworks 5 years later. Child Development, 84(5), 1526–1541. 10.1111/cdev.12064, 23397904 PMC3655123

[bib35] Gunderson, E. A., Sorhagen, N. S., Gripshover, S. J., Dweck, C. S., Goldin-Meadow, S., & Levine, S. C. (2018). Parent praise to toddlers predicts fourth grade academic achievement via children’s incremental mindsets. Developmental Psychology, 54(3), 397–409. 10.1037/dev0000444, 29172567 PMC5826820

[bib36] Guo, J., Hu, X., Elliot, A. J., Marsh, H. W., Murayama, K., Basarkod, G., Parker, P. D., & Dicke, T. (2023). Mastery-approach goals: A large-scale cross-cultural analysis of antecedents and consequences. Journal of Personality and Social Psychology, 125(2), 397–420. 10.1037/pspp0000436, 36136789

[bib37] Gweon, H., & Schulz, L. (2019). From exploration to instruction: Children learn from exploration and tailor their demonstrations to observers’ goals and competence. Child Development, 90(1), e148–e164. 10.1111/cdev.13059, 29635785

[bib38] Gweon, H., Shafto, P., & Schulz, L. (2018). Development of children’s sensitivity to overinformativeness in learning and teaching. Developmental Psychology, 54(11), 2113–2125. 10.1037/dev0000580, 30265027

[bib39] Harris, A., Yuill, N., & Luckin, R. (2008). The influence of context-specific and dispositional achievement goals on children’s paired collaborative interaction. British Journal of Educational Psychology, 78(3), 355–374. 10.1348/000709907X267067, 18086339

[bib40] Henrich, J., Heine, S. J., & Norenzayan, A. (2010). Most people are not WEIRD. Nature, 466(7302), 29. 10.1038/466029a, 20595995

[bib41] Heyman, G. D., & Dweck, C. S. (1992). Achievement goals and intrinsic motivation: Their relation and their role in adaptive motivation. Motivation and Emotion, 16(3), 231–247. 10.1007/BF00991653

[bib42] Huang, C. (2012). Discriminant and criterion-related validity of achievement goals in predicting academic achievement: A meta-analysis. Journal of Educational Psychology, 104(1), 48–73. 10.1037/a0026223

[bib43] Hulleman, C. S., Schrager, S. M., Bodmann, S. M., & Harackiewicz, J. M. (2010). A meta-analytic review of achievement goal measures: Different labels for the same constructs or different constructs with similar labels? Psychological Bulletin, 136(3), 422–449. 10.1037/a0018947, 20438145

[bib44] Jeong, J., & Frye, D. A. (2025). Approximating the ZPD? Young children’s judgements of appropriate task level for learning. British Journal of Developmental Psychology, 43(1), 37–65. 10.1111/bjdp.12519, 39193835

[bib45] Leonard, J. A., Cordrey, S. R., Liu, H. Z., & Mackey, A. P. (2023). Young children calibrate effort based on the trajectory of their performance. Developmental Psychology, 59(3), 609–619. 10.1037/dev0001467, 36174183

[bib46] Leonard, J. A., & Sommerville, J. A. (2025). A unified account of why optimism declines in childhood. Nature Reviews Psychology, 4(1), 35–48. 10.1038/s44159-024-00384-z

[bib47] Maaz, K., Trautwein, U., Lüdtke, O., & Baumert, J. (2008). Educational transitions and differential learning environments: How explicit between-school tracking contributes to social inequality in educational outcomes. Child Development Perspectives, 2(2), 99–106. 10.1111/j.1750-8606.2008.00048.x

[bib48] Mageau, G. A., Bureau, J. S., Ranger, F., Allen, M.-P., & Soenens, B. (2016). The role of parental achievement goals in predicting autonomy-supportive and controlling parenting. Journal of Child and Family Studies, 25(5), 1702–1711. 10.1007/s10826-015-0341-1

[bib49] Magid, R. W., DePascale, M., & Schulz, L. E. (2018). Four- and 5-year-olds infer differences in relative ability and appropriately allocate roles to achieve cooperative, competitive, and prosocial goals. Open Mind: Discoveries in Cognitive Science, 2(2), 72–85. 10.1162/opmi_a_00019

[bib50] Meece, J. L., Blumenfeld, P. C., & Hoyle, R. H. (1988). Students’ goal orientations and cognitive engagement in classroom activities. Journal of Educational Psychology, 80(4), 514–523. 10.1037/0022-0663.80.4.514

[bib51] Midgley, C., Kaplan, A., & Middleton, M. (2001). Performance-approach goals: Good for what, for whom, under what circumstances, and at what cost? Journal of Educational Psychology, 93(1), 77–86. 10.1037/0022-0663.93.1.77

[bib52] Newall, C., Gonsalkorale, K., Walker, E., Forbes, G. A., Highfield, K., & Sweller, N. (2018). Science education: Adult biases because of the child’s gender and gender stereotypicality. Contemporary Educational Psychology, 55, 30–41. 10.1016/j.cedpsych.2018.08.003

[bib53] Nussenbaum, K., Martin, R. E., Maulhardt, S., Yang, Y. J., Bizzell-Hatcher, G., Bhatt, N. S., Koenig, M., Rosenbaum, G. M., O’Doherty, J. P., Cockburn, J., & Hartley, C. A. (2023). Novelty and uncertainty differentially drive exploration across development. eLife, 12, e84260. 10.7554/eLife.84260, 37585251 PMC10431916

[bib54] Ornstein, A. C. (1997). How teachers plan lessons. High School Journal, 80(4), 227–237.

[bib55] Pintrich, P. R. (2003). A motivational science perspective on the role of student motivation in learning and teaching contexts. Journal of Educational Psychology, 95(4), 667–686. 10.1037/0022-0663.95.4.667

[bib56] Robinson, K. A. (2023). Motivational climate theory: Disentangling definitions and roles of classroom motivational support, climate, and microclimates. Educational Psychologist, 58(2), 92–110. 10.1080/00461520.2023.2198011

[bib57] Rosenthal, R., & Jacobson, L. (1968). Pygmalion in the classroom. Urban Review, 3(1), 16–20. 10.1007/BF02322211

[bib58] Rubovits, P. C., & Maehr, M. L. (1973). Pygmalion black and white. Journal of Personality and Social Psychology, 25(2), 210–218. 10.1037/h0034080

[bib59] Ruggeri, A. (2022). An introduction to ecological active learning. Current Directions in Psychological Science, 31(6), 471–479. 10.1177/09637214221112114

[bib61] Scherrer, V., Preckel, F., Schmidt, I., & Elliot, A. J. (2020). Development of achievement goals and their relation to academic interest and achievement in adolescence: A review of the literature and two longitudinal studies. Developmental Psychology, 56(4), 795–814. 10.1037/dev0000898, 32052983

[bib62] Schiefele, U., & Schaffner, E. (2015). Teacher interests, mastery goals, and self-efficacy as predictors of instructional practices and student motivation. Contemporary Educational Psychology, 42, 159–171. 10.1016/j.cedpsych.2015.06.005

[bib63] Schneider, W. (1998). Performance prediction in young children: Effects of skill, metacognition and wishful thinking. Developmental Science, 1(2), 291–297. 10.1111/1467-7687.00044

[bib64] Senko, C. (2016). Achievement goal theory: A story of early promises, eventual discords, and future possibilities. In K. R. Wentzel & D. B. Miele (Eds.), Handbook of motivation at school (2nd ed., pp. 75–95). Routledge. 10.4324/9781315773384-6

[bib65] Senko, C., & Dawson, B. (2017). Performance-approach goal effects depend on how they are defined: Meta-analytic evidence from multiple educational outcomes. Journal of Educational Psychology, 109(4), 574–598. 10.1037/edu0000160

[bib66] Serko, D., Leonard, J., & Ruggeri, A. (2025). Children strategically decide what to practice. Child Development, 96(5), 1619–1631. 10.1111/cdev.14268, 40448508 PMC12379865

[bib67] Shachnai, R., Belluck, A., & Leonard, J. A. (2025). Parents underestimate young children’s abilities which may undermine their parenting practices. In D. Barner, N. R. Bramley, A. Ruggeri, & C. M. Walker (Eds.), Proceedings of the 47th Annual Conference of the Cognitive Science Society (pp. 5817–5824). Cognitive Science Society.

[bib68] Shim, S. S., Cho, Y., & Cassady, J. (2013). Goal structures: The role of teachers’ achievement goals and theories of intelligence. Journal of Experimental Education, 81(1), 84–104. 10.1080/00220973.2011.635168

[bib69] Shutts, K., Brey, E. L., Dornbusch, L. A., Slywotzky, N., & Olson, K. R. (2016). Children use wealth cues to evaluate others. PLOS ONE, 11(3), e0149360. 10.1371/journal.pone.0149360, 26933887 PMC4774995

[bib70] Sierksma, J., & Shutts, K. (2020). When helping hurts: Children think groups that receive help are less smart. Child Development, 91(3), 715–723. 10.1111/cdev.13351, 31900939 PMC7244365

[bib71] Sobel, D. M., & Letourneau, S. M. (2015). Children’s developing understanding of what and how they learn. Journal of Experimental Child Psychology, 132, 221–229. 10.1016/j.jecp.2015.01.004, 25728930

[bib72] Strauman, T. J., & Wilson, W. A. (2010). Individual differences in approach and avoidance: Behavioral activation/inhibition and regulatory focus as distinct levels of analysis. In R. H. Hoyle (Ed.), Handbook of personality and self-regulation (pp. 447–473). John Wiley & Sons, Ltd. 10.1002/9781444318111.ch20

[bib73] Tenenbaum, H. R., & Ruck, M. D. (2007). Are teachers’ expectations different for racial minority than for European American students? A meta-analysis. Journal of Educational Psychology, 99(2), 253–273. 10.1037/0022-0663.99.2.253

[bib74] van der Veer, R., & Valsiner, J. (1991). Understanding Vygotsky: A quest for synthesis. Blackwell Publishing.

[bib75] Vygotsky, L. S. (1978). Mind in society: Development of higher psychological processes. Harvard University Press. 10.2307/j.ctvjf9vz4

[bib76] Walker, C. M., & Gopnik, A. (2014). Toddlers infer higher-order relational principles in causal learning. Psychological Science, 25(1), 161–169. 10.1177/0956797613502983, 24270464

[bib77] Wass, R., & Golding, C. (2014). Sharpening a tool for teaching: The zone of proximal development. Teaching in Higher Education, 19(6), 671–684. 10.1080/13562517.2014.901958

[bib78] Woodcock, A., Hernandez, P. R., & Schultz, P. W. (2016). Diversifying science: Intervention programs moderate the effect of stereotype threat on motivation and career choice. Social Psychological and Personality Science, 7(2), 184–192. 10.1177/1948550615608401, 27668075 PMC5034946

[bib79] Yeager, D. S., Hanselman, P., Walton, G. M., Murray, J. S., Crosnoe, R., Muller, C., Tipton, E., Schneider, B., Hulleman, C. S., Hinojosa, C. P., Paunesku, D., Romero, C., Flint, K., Roberts, A., Trott, J., Iachan, R., Buontempo, J., Yang, S. M., Carvalho, C. M., … Dweck, C. S. (2019). A national experiment reveals where a growth mindset improves achievement. Nature, 573(7774), 364–369. 10.1038/s41586-019-1466-y, 31391586 PMC6786290

[bib80] Zhang, X., Carrillo, B. A., Christakis, A., & Leonard, J. A. (2025). Children predict improvement on novel skill learning tasks. Child Development, 96(3), 1177–1188. 10.1111/cdev.14232, 40171764

