# Supplementary material for: Children Understand How Adults’ Achievement Goals Drive Actions
Source: Open Mind (Camb). 2026 Feb 10;10:160–81. doi: 10.1162/OPMI.a.331 (PMC13053020; doi:10.1162/OPMI.a.331)
Supplement: Supplementary file 1 [file opmi-10-160-s001.pdf]

0. Summary

Supplementary materials for “Children understand how adults’ achievement goals drive actions”. These materials contain supplementary materials, regression tables, and results for Experiments 1 (A-C) and 2 (A-B).

1. Experiment 1

1.1 Pilot for Experiment 1

Twenty-two 5- to 6-year-old participants (M_{Age} (SD) = 71.86 (7.36) months; 45% female; 73% White) were asked to compare the tracing abilities between a child who was the “exact same age” as them (i.e., 5- or 6-years-old) and a 10-year-old child. Participants were shown a static image of two illustrated children where one child’s image was larger than the other child’s to denote who was older (see SM Figure 1). Participants were then asked, “Who do you think is better at tracing?”, to which they could respond with the color of the friend’s shirt (e.g., “yellow” or “red”). In total, 18 of 22 participants (82%, $p = 0.002$, binomial test against 50% chance) said the 10-year-old child was better at tracing. We used these results when deciding to make age a proxy for child competence for Experiments 1A-C.

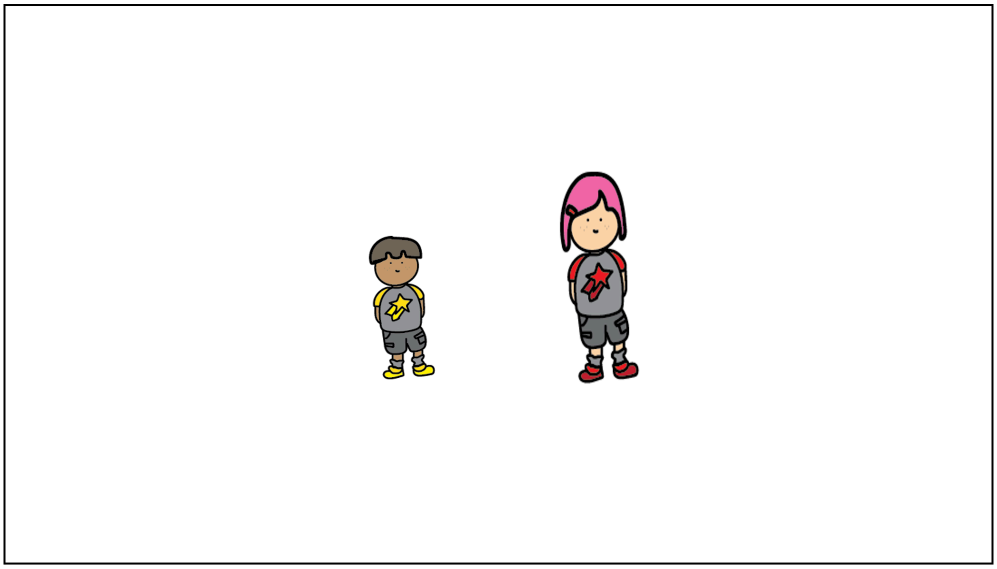

SM Figure 1. Tracing competence comparison slide. Participants saw this image and were asked to decide who was more competent at tracing between the age-matched child (yellow) and the 10-year-old child (red).

1.2 Experiment 1A

1.2.1 Supplementary Results

Regressions within achievement goal trials (preregistered)

Participants were more likely to choose harder tracings for a more competent target child versus a less competent target child across both learning goal trials (b = −3.67, 95% CI [-8.05, -1.55]) and performance goal trials (b = −1.99, 95% CI [-5.39, -0.24]).

Regressions within child competence trials (preregistered)

Participants were more likely to choose harder tracings when holding a learning goal over a performance goal for a less competent target child (b = −5.22, 95% CI [-11.59, -2.17]) and a more competent target child (b = −6.00, 95% CI [-13.79, -2.08]).

Chi-square tests (exploratory)

The distribution of participants' tracing choices were significantly different from chance (33%) across both performance goal trials (less competent target child trial: χ^2 (2,40) = 53.15, p < 0.001; more competent target child trial: χ^2 (2,40) = 18.2, p < 0.001) and both learning goal trials (less competent target child trial: χ^2 (2,40) = 21.05, p < 0.001; more competent target child trial: χ^2 (2,40) = 24.05, p < 0.001).

1.3 Experiment 1B

1.3.1 Supplementary Results

Regressions within achievement goal trials (preregistered)

Participants were not more likely to predict that adults would give harder tracings to more competent, 10-year-old children than to less competent, age-matched children in the performance goal trials (b = 0.94, 95% CI [-0.21, 3.22]) or the learning goal trials (b = 1.10, 95% CI [-0.21, 3.91]). These findings could suggest that children's task selection predictions are weakly sensitive to a target child's competence.

Regressions within child competence trials (preregistered)

Participants were more likely to predict that an adult would choose a harder level of tracing when the adult had a learning goal versus a performance goal in the less competent target child trials (b = −1.62, 95% CI [-4.85, -0.10]) and in the more competence target child trials (b = −1.71, 95% CI [-4.35, -0.25]).

Chi-square tests (exploratory)

The distribution of participants' tracing choices were significantly different from chance (33%) across both performance goal trials (less competent target child trial: χ^2 (2,40) = 6.05, p = 0.05; more competent target child trial: χ^2 (2,40) = 6.95, p = 0.03) and the learning goal trial when the target child was more competent (χ^2 (2,40) = 6.35, p = 0.04) but not when the target child was less competent (χ^2 (2,40) = 0.8, p = 0.67).

Binomial tests (exploratory)

Participants did not selectively predict any of the tracings in the learning goal trial when the target child was less competent (easy: p = 1; medium: p = 1; hard: p = 1; all reported p-values are with Bonferroni corrections) or when the target was more competent (easy: p = 1; medium: p = 1; hard: p = 0.08). Participants did not selectively predict any of the tracings in the performance goal trial when the target child was less competent (easy: p = 0.72; medium: p = 0.72; hard: p = 0.05). Participants did, however, selectively predict the medium tracing (p = 0.03) – and not the easy (p = 1) or hard (p = 0.28) tracing – for a performance goal when the target child was more competent.

Analyses including excluded participants (exploratory)

Sixteen participants were excluded in Experiment 1B. Of these 16 exclusions, 14 were excluded due to failing to answer more than half (i.e., 3-4 out of 4 total) of the memory check questions asked across the four test trials (the other 2 were excluded for failing to complete the experiment). We re-ran our main additive model with these 14 children included and our results remained the same: Children were more likely to predict an adult would choose easier tracings

when that adult had a performance (vs. learning) goal ($b = -0.78$, 95% CI [-1.35, -0.25]) and when the child receiving the tracing was more (vs. less) competent ($b = 0.50$, 95% CI [0.14, 0.86]. This suggests that our results are not driven by children with better memory or verbal abilities.

1.4 Experiment 1C

1.4.1 Supplementary Materials

Example of stimuli used in "Receive" trials

In Experiment 1C, 5- and 6-year-old participants were told that two of the experimenter's friends had chosen tracings for them before the study began. When participants were asked to pick the tracing they believed the friend chose for them, participants were shown an image of a silhouetted adult figure (see SM Figure 2).

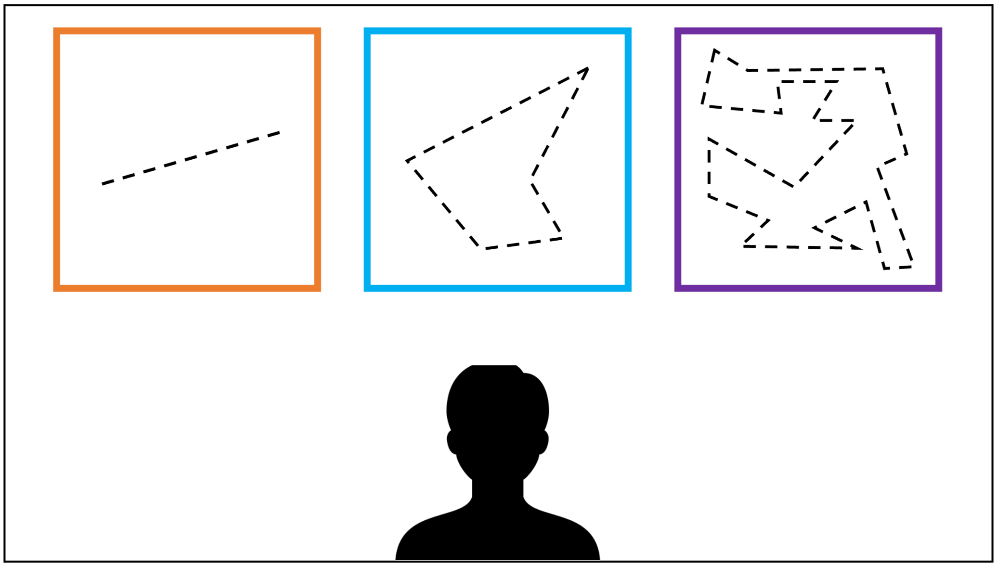

SM Figure 2. Example of a "receive" trial. The silhouetted figure is meant to represent one of the experimenter's "friends" who had previously chosen a tracing for the participant.

1.4.2 Supplementary Results

Regressions within achievement goal trials (preregistered)

There was no difference between participants' predictions of which tracing they would receive and what they chose themselves in either the performance (b = 0.43, 95% CI [-0.90, 2.15]) or learning goal trials (b = 0.05, 95% CI [-1.51, 1.77]).

Regressions within child role trials (preregistered)

Participants were more likely to choose a harder difficulty tracing when the "giver" had a learning goal in both the receive trials (b = −2.16, 95% CI [-5.81, -0.30]) and in the choose trials (b = −2.79, 95% CI [-6.86, -0.69]).

Chi-square tests (exploratory)

The distribution of participants' tracing choices was only marginally different from chance (33%) when holding a performance goal and choosing for themselves (χ^2 (2,40) = 5.15, p = 0.08), and not significant different from chance when predicting what someone else chose for them (χ^2 (2,40) = 1.85, p = 0.4). However, the distribution of participants tracing choices were significantly different from chance across both learning goal trials, both when choosing for themselves (χ^2 (2,40) = 6.35, p = 0.04) and predicting what someone else chose for them (χ^2 (2,40) = 6.65, p = 0.04).

Binomial tests (exploratory)

Participants did not selectively predict they would receive any particular tracing in either the performance (easy: p = 0.72; medium: p = 1; hard: p = 0.95) or the learning (easy: p = 0.051; medium: p = 1; hard: p = 0.19) goal trials. Participants did not selectively choose a tracing for themselves in either the performance (easy: p = 0.08; medium: p = 1; hard: p = 0.54) or the learning (easy: p = 0.13; medium: p = 1; hard: p = 0.08) goal trials as well.

2. Experiment 2

2.1 Experiment 2A

2.1.1 Supplementary Materials

Reading Level tracker

We created a "Reading Level tracker" to help participants think about both a target child's specific competence level and the granularity of their own task choices (see SM Figure 3).

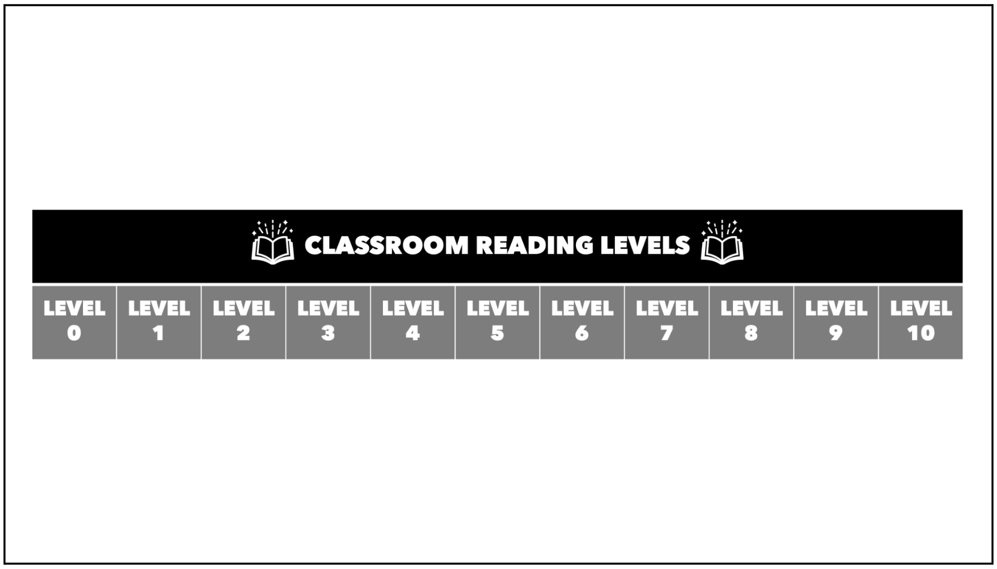

SM Figure 3. The Reading Level "tracker" participants were introduced to in Experiment 2A-B. Reading Levels ranged from Level 0 (easiest difficulty) to Level 10 (hardest difficulty).

2.1.2 Supplementary Results

Regressions within achievement goal and child competence trials (preregistered)

Participants were more likely to choose higher levels when they possessed a learning goal (vs. a performance goal) for each competence level (b's $\geq$ 1.48, p's < 0.001) and more likely to choose harder tracings for more competent children in each goal condition (b's $\geq$ 1.94, p's < 0.001)

2.2 Experiment 2B

2.2.1 Supplementary Materials

Comprehension checks

To ensure children in our experiment understood task difficulty and child competence relative to the "Reading Level" scale, we asked a series of comprehension check questions at the beginning of each experiment. First, children were asked to state which level was the easiest (i.e., Level 0) and which level was the hardest (i.e., Level 10; all children passed). Next, children were shown a Reading Level 5 student in orange and a Reading Level 7 student in blue, then shown a book from Reading Level 6. Children were asked to say which student they believed would have an easier time reading the book from Level 6 (in this case, the student with a higher Reading Level, as opposed to the student with a lower level, would find the Level 6 book easier to read). If any child missed this comprehension check question, the experimenter corrected them and reminded them of each student's Reading Level. If a child missed this question a second time, their data was excluded from our analyses (n = 4). Lastly, children were again shown the Reading Level 5 student and were asked whether the student could read a book from Level 0 and Level 10 perfectly. No children were excluded based on their responses. The majority of children thought a Reading Level 5 student could read a Level 0 book perfectly (n = 79/80 responded "yes" when asked, 99%) but could not read a Level 10 book perfectly (n = 79/80 responded "no" when asked, 99%).

Additionally, to ensure children understood the phrasing of the performance goal used in the experiment (i.e., "read a book perfectly in order to earn a sticker"), we included two more comprehension check questions after the ones described previously. Children were shown two students who both read a book out loud. One student read the book "perfectly with no mistakes" (indicated by a green check mark), while the other student did not read the book perfectly and "made mistakes" (indicated by a red X). Children were asked whether each student would earn a sticker. If children failed to accurately state whether a student would or would not earn a sticker, they were corrected by the experimenter. No children in our sample missed these performance goal comprehension checks.

2.2.3 Supplementary Results

Comparison between performance goal trials (preregistered)

When looking at children's teacher choices within the two performance goal trials, our logistic mixed-effects regression revealed a main effect of trial (b = 11.65, p < 0.001) but no main effect of participant age (b = -0.01, p = 0.44). These results suggest that children were more likely to select the teacher who made the most optimal task choice (i.e., a Level 4 book) in the Level 4 vs. Level 6 trial compared to the Level 2 vs. Level 4 trial.

Comparison between learning goal trials (preregistered)

When looking at children's teacher choices within the two learning goal trials, our model found a marginal effect of trial (b = -9.28, p = 0.09) and a main effect of participant age (b = 0.09, p < 0.001). To further interrogate this potential age effect, we ran a separate logistic interaction regression (same model described above but with an interaction term). We did, indeed, find a significant interaction (b = 0.76, p = 0.02). These findings show that children were more likely to choose the teacher who made the most optimal task choice (i.e., a Level 6 book) in the Level 4 vs. 6 trial compared to the Level 6 vs. 8 trial. However, younger children in our sample were more likely to show this pattern of response than older children. Older children were more likely to choose the teacher who chose a Level 6 book as the adult best satisfying their learning goal across *both* learning goal trials.

Analyses including excluded participants (exploratory)

Twenty-three participants were excluded in Experiment 2B. Of these 23 exclusions, 15 were excluded either for failing comprehension check questions or for experimenter/technical errors, but still had full response data (the other 8 had incomplete data due to failing to complete the experiment, n = 4, or experimenter/technological error, n = 4). As in Experiment 1B, after running our preregistered additive model with these 16 children included, we found our results remained the same: Children chose the Level 6 book teacher more than the Level 4 book teacher when the teachers possessed learning goals versus performance goals (b = -2.52, p < 0.001). When both teachers possessed a learning goal, the majority of children (n = 68/95; 72%) said the teacher who chose a Level 6 book better satisfied the goal (p < 0.001, binomial test against chance, 50%). However, when both teachers possessed a performance goal, the majority of children (n = 68/95; 72%) said the teacher who chose a Level 4 book better satisfied the goal (p < 0.001, binomial test against chance, 50%). This analysis suggests that our key results are not driven by children with strong memory or verbal abilities.

3. Table of Unstandardized and Standardized Model Estimates

Experiment (Result)	Unstandardized Estimate (*B*)	Standardized Estimate (β)
Experiment 1A (main effect of achievement goal)	*B* = -3.76*	β = -3.76*
Experiment 1A (main effect of child competence)	*B* = 2.00*	β = 2.00*
Experiment 1B (main effect of achievement goal)	*B* = -0.82*	β = -0.82*
Experiment 1B (main effect of child competence)	*B* = 0.46*	β = 0.46*
Experiment 1C (main effect of achievement goal)	*B* = -2.69*	β = -2.69*
Experiment 1C (main effect of participant role)	*B* = -0.21	β = -0.21
Experiment 2A (main effect of achievement goal)	*B* = 1.58*	β = 3.19*
Experiment 2A (main effect of child competence) [Reading Level 5]	*B* = 1.99*	β = 4.02*
Experiment 2A (main effect of child competence) [Reading Level 7]	*B* = 3.88*	β = 7.84*
Experiment 2B (main effect of achievement goal) [Level 4 vs. Level 6 trials]	*B* = -2.89*	β = -1.60*
Experiment 2B (main effect of participant age) [Level 4 vs. Level 6 trials]	*B* = 0.02	β = 0.24

SM Table 1. Calculation of standardized estimates. For Experiment 1A-C, since we used a standard normal latent variable for modeling ordinal data (i.e., "probit" model) and this latent variable has a SD of 1, we divide our estimates by 1 to achieve a standardized estimate. For Experiment 2A, we divide the unstandardized coefficients by the model's residual standard deviation (sigma) to achieve a standardized estimate. To achieve a standardized estimate for Experiment 2B, we multiplied the unstandardized estimates by ($\sqrt{3}/\pi$) (as appropriate for a logistic regression, see Nakagawa & Cuthill, 2007; Borenstein et al., 2009) for any factorized predictors. However, to achieve a standardized estimate for factors that were numeric, we multiplied the unstandardized estimates by their standard deviation.

* $p < 0.05$ or 95% CI does not cross zero
